# Supplementary material for: Integrative analysis of ATAC-seq and RNA-seq for cells infected by human T-cell leukemia virus type 1
Source: PLoS Comput Biol. 2025 Jan 2;21(1):e1012690. doi: 10.1371/journal.pcbi.1012690 (PMC11753684; doi:10.1371/journal.pcbi.1012690)
Supplement: Attachment — Submitted filename: Response_to_reviewers_240906.pdf [file pcbi.1012690.s002.pdf]

Dear reviewers,

Thank you very much for providing us the constructive feedback regarding our manuscript. Following the comments, we hereby resubmit our revised manuscript.
Thank you once again for your consideration of our paper.

Sincerely,

Azusa Tanaka, Yasuhiro Ishitsuka, Hiroki Ohta, Norihiro Takenouchi, Masanori Nakagawa, Ki-Ryang Koh, Chiho Onish, Hiromitsu Tanaka, Akihiro Fujimoto, Jun-ichirou Yasunaga, Masao Matsuoka

First, we would like to explain that we have found some errors in our calculation in our previous manuscript, and then, through correcting the errors, we have made significant improvement, which is included in this major revision, as follows.

In the previous version of our manuscript, as Fig.7 (the previous version), we tried to plot the histogram of log-fold change of gene expression averaged over samples between two different cell types, by focusing on only the genes selected by our previously developed clustering algorithm.

Indeed, a set of the selected genes depends on a given pair of two cell types.
Relate to this, we made the following mistake; We detected one set of the selected genes for the pair of CD4+T and ATL, which we used for all the case of the pairs in addition to the case of CD4+T and ATL. Thus, the histogram for the pairs in Fig. 7 (the previous version) except for CD4+T and ATL was not what we tried to see.

After using the correctly selected genes depending on the pair of two cell types, we realized that this method is not very proper to capture characteristic properties inherent to ATL cells.

Instead of correctly performing the previous analysis, we have focused on the correlation between chromatin accessibility quantified by ATAC-seq and the gene expression quantified by RNA-seq. Concretely, we have analyzed, for a given pair of two cell types, the log-fold change of peak width of open chromatin regions for a given gene i and also the gene expression of gene i.
As shown in Fig. 7, we have plotted the scatter plot where the horizontal axis "x" corresponds to that of the peak width, and the vertical axis "y" corresponds to that of the gene expression. Further, we have also plotted the two-dimensional histogram in terms of the polar coordinate system (R, θ) for (x, y)-coordinate system, where $x = R\cos\theta$ and $y = R\sin\theta$.

It has turned out that this two-dimensional histogram of the polar coordinate system is useful to capture unique properties in ATL cases, which we discussed in detail in the main text. Thus, we have replaced the previous version of Fig. 7 by the two-dimensional histograms for some pairs of two cell types, as shown in Fig.7 and Fig. 8.

Further, as the second referee pointed out, we have uploaded our codes and data to produce Fig. 4, Fig.5, Fig. 7, Fig.8, and Fig. 11, for which we would need rather careful explanation of how to calculate, in the website (link: https://osf.io/6gtuy/). Note that the code to produce Table 2 was already uploaded in the website (link: https://github.com/tanakanishi/findclosest) when [Tanaka, et al. PLOS Comput. Biol. 2020] was published. All the data used in this project are deposited at the DNA Data Bank of Japan (DDBJ) under accession numbers PRJDB18166 and PRJDB18259.
****

In the following, we reply to the referee's comments and explain the corresponding revisions.

Reviewer #1:
Tanaka and colleagues studied about the relationship between chromatin accessibility and pathogenesis of ATL and HAM, especially ATL. It is interesting work but first round reviewers pointed out many concerns. The authors should reply carefully to the comments.

Comment A-1)
In comment 1-2, the referee pointed out the lack of biological considerations and new discoveries about ATL /HAM, but the authors replied by the comments about CTCL. They should reply about the relevance concerning ATL/HAM. Moreover, in the discussion about CTCL, the relationship between romidepsin treatment responsiveness and pre-treatment chromatin structure is unclear.

Reply A-1)
We were not completely sure about what biological consideration and new discoveries in the previous referee comment mean. In this work throughout, we have already shown new discoveries for HAM and ATL samples, which have been obtained from the analysis of biological data based on several aspects of chromatin level, gene expression level, and protein level. Then, in the section of discussion, we have discussed the promising future work about HAM and ATL based on our findings in this paper.

Since we do not have enough data of symptom of ATL and HAM, we intended to discuss ATAC-seq of CTCL to investigate the universality of the finding in ATL in other leukemia samples. Nevertheless, we agree that it was not clear as the referee pointed out. Then, we have decided to focus on only the classification of CTCL samples without considering treatment. Note that it has been well-known that ATL and HAM have a couple of decades incubation period, which makes it difficult to collect enough data for the analysis of the

symptoms.

As we have already mentioned, the part of the analysis of CTCL data is not main result of this paper, which can be removed without loss of reliability of the main results. We have an option to do it if it is potentially confusing to the readers.

Revision A-1)

In order to clarify the part of the analysis of CTCL data, we have revised the corresponding part with emphasis on the classification of the ATAC-seq of some CTCL cases as myeloid cells.

Comment A-2)

In comment 1-3, the referee commented about significance of chromatin conformational changes for the disease, but your reply did not refer to the point. Further 3d structural changes were not out of scope in this comment.

Reply A-2)

We have found that ATAC-seq data has information which make us find TLL1 as a gene showing anomalous expression, which is one of the main results in this paper. However, we have not yet investigated how TLL1 affects the disease from the aspect of symptom, which remains as an intriguing future study.

As already replied in the previous communication, ATAC-seq data that we used in this paper do not have full resolution of 3d structural change of the chromatin. Therefore, we could not go into this topic.

Revision A-2)

In the section of discussion, we have added the sentences "Thus, we found that ATAC-seq data had information which leads us to find TLL1 as a gene showing important gene expression. However, we have not yet investigated how TLL1 affects the disease from the aspect of symptom, which remains as an intriguing future study."

Comment A-3)

In comment 1-4, the authors should reply about overall expression pattern such as heatmap. Revision 1-4 did not reply to the referee’s comment.

Reply A-3)

Following the comment, we have shown an example of heatmap of overall expression pattern as Fig. 6a.

Comment A-4)
The expression level of TLL1 isoform 1 is about 10.3 times higher than that of TLL isoform 2. The authors should show the data.

Reply A-4)
We have added the data of 4 samples as well as the average in Table 3, from which we have calculated that quantity in the corresponding part.

Comment A-5)
As pointed out in comment 1-5, chromatin conformational changes in TLL1 have also been observed in Fig 10B. The referee asked the authors to consider whether the effects of TGF are also likely to be affected in HAM, but there was no consideration in the authors' revision.

Reply A-5)
As we have already mentioned, we do not have enough data of HAM cases. Therefore, we could not have reliable consideration about it. Even though, following the referee's comment, in the section of discussion, we have added the sentence of "due to the lack of proper RNA-seq data of HAM samples, we were unable to study gene expression of HAM samples such as effects to TGF-β, which remain to be analyzed in future studies".

Comment A-6)
As pointed out in comment1-6, I also think that footprint and other data should be moved to the results section and discussed in the discussion section.

Reply A-6)
We have made the parts of footprint move to section 2-F and the gene expression in ATL cells move to section 2-H.

Comment A-7)
It is not clear that remidepsin treatment responsiveness has relationship with pre-treatment chromatin structure from the data shown in the manuscript.

Reply A-7)
As we have already replied in Reply A-1), we agree that this part was not clear. Then, we have made revision for it as described in Revision A-1).

Reviewer #2:

This study investigates the interplay between chromatin accessibility and transcription in HTLV-1-infected cells at a genome-wide scale, utilizing ATAC-seq and RNA-seq data. By employing a systematic clustering algorithm, the authors compared HTLV-1 infected CD4+ cells from ATL and HAM cases with healthy CD4+ T cells. They observed abnormal properties in the open chromatin regions of CD4+ T cells derived from ATL cases compared to healthy counterparts, along with distinct gene expression patterns between the two groups based on immunophenotype. Furthermore, the analysis revealed a relationship between chromatin accessibility and immunophenotype, indicating a potential resemblance of some ATL cases to various myeloid cell types. Notably, the study identified TLL1 as one of the genes with aberrant expression in ATL cases, with subsequent experiments demonstrating isoform-dependent regulation of TGF-β maturation by TLL1 isoforms, which is relevant to cancer progression. This study is well-designed and has uncovered novel findings, shedding light on previously unexplored aspects of ATL. I have a few minor comments.

Comment B-1)

HTLV-1 infected CD4+ cells are thought to be consisted mainly of leukemic cells in ATL. If so, it would be better to clearly state this.

Reply B-1)

HTLV-1-infected CD4+ cells in this paper operationally mean the cells showing CADM1 positive and CD4 positive, which are thought to be consisted mainly of leukemic cells in ATL and HAM cases. The detail of this experimental operation is described in Materials and Methods A.

Revision B-1)

As the referee has pointed out, we have inserted the sentence of "HTLV-1-infected CD4+ cells in this paper operationally mean the cells showing CADM1 positive and CD4 positive, which are thought to be consisted mainly of leukemic cells in ATL and HAM cases." in the second paragraph of section 2-A.

Comment B-2)

It would be better to make discussions based on more recent studies using human ATL and HAM samples. For example, there have been several papers investigating i) epigenetic status using ChIP-seq and methylation microarray, ii) non-coding genome using whole-genome sequencing, and iii) immunophenotypes and heterogeneity (or fluctuations) within and among patients using single-cell analysis in ATL.

Reply B-2)

As the referee has pointed out, we have cited [Yamagishi, et al. Nature 2024] and [Koseki, et al. Front. Microbiol. 2023] for topic i), [Kogure, et al. Blood 2022] for topic ii), and [Koya, et al. Blood Cancer Discov. 2021] for topic iii). Then, we have made discussion about the relevance of such recent studies to the results found in our work.

Revision B-3)

As the 7th and 8th paragraphs in the section of discussion, we have inserted the corresponding paragraphs.

Comment B-3)

Gene name should be italicized.

Reply B-3)

We have made all the gene names italicized.

We would like to again thank the referees for the comments that have helped us improve the paper.
