# Supplementary material for: Integrative analysis of ATAC-seq and RNA-seq for cells infected by human T-cell leukemia virus type 1
Source: PLoS Comput Biol. 2025 Jan 2;21(1):e1012690. doi: 10.1371/journal.pcbi.1012690 (PMC11753684; doi:10.1371/journal.pcbi.1012690)
Supplement: Attachment — Submitted filename: Reply_241015.pdf [file pcbi.1012690.s003.pdf]

Dear reviewers,

Thank you very much for your constructive comments and accurate suggestions on our manuscript. Following the comments and suggestions, we hereby resubmit our revised manuscript.
Thank you once again for your consideration of our paper.

Sincerely,

Azusa Tanaka, Yasuhiro Ishitsuka, Hiroki Ohta, Norihiro Takenouchi, Masanori Nakagawa, Ki-Ryang Koh, Chiho Onish, Hiromitsu Tanaka, Akihiro Fujimoto, Jun-ichirou Yasunaga, Masao Matsuoka

****************************

Reviewer #1:
The manuscript is well-revised and now worth to be published.
I have some minor comments and question. Please consider minor modification.

Comment 1-1)
This study analyzed the peripheral blood sample of ATL and HAM patients, not asymptomatic carriers. So, the result of this study is not necessarily reflect the change caused by HTLV-1 infection alone. The authors should be careful to this point.

Also, to analyze aggressive ATL cells, CADM1+/CD7- fraction should be analyzed. CD4+/CADM1+ fraction of the peripheral blood of ATL patients include true aggressive tumor cells and those which are HTLV-1 infected but not fully transformed.

Reply 1-1)
As the referee commented, without analyzing the carrier samples, we cannot say for sure whether the changes are due to HTLV1 infection or not. In this study, we analyzed three carrier samples and found that the TLL1 region was open even in carriers. Therefore, this region is presumed to be involved in HTLV1 infection. However, genome-wide analysis requires an increase in the number of cases, which is an issue to be addressed in the future.

In our paper, only CD4+CADM1+ cells were analyzed, but we have already obtained single-cell multiome (ATAC+RNAseq) data and would like to focus on the expression of CD7 in future analyses.

Revision 1-1)
Corresponding to the former part of the comment, "carriers" in Fig. 12b was not mentioned in the main text. Therefore, we have revised a part in 10th paragraph of section "Discussion" as follows:

The phrase "HAM cases" is replaced by the phrase "the case of HAM and HTLV-1 carrier (carrier)".

Comment 1-2)

In section F. I cannot understand why it is considered that HTLV-1 infected cells have already differentiated into T cells before infection. Is the possibility that HTLV-1 infected cell differentiated into T cell and TCR rearrangement occur after infection is excluded?

Reply 1-2)

We appreciate this important comment. As the referee pointed out, the only thing that can be said from this data is that at the time the ATL/HAM samples were collected, the HTLV-1 infected cells had differentiated into T cells. Therefore, we have revised this part as follows.

Revision 1-2)

At the end of the third paragraph in section 2-F, where the following sentence was written:

"Thus, this is a supporting evidence from the viewpoint of the chromatin level that the infected cells had already differentiated into T cells before infection."

We have replaced it by the following sentence:

"Thus, this is supporting evidence, in terms of chromatin levels, that the infected cells of the ATL/HAM patients used in the analysis were differentiated into T cells."

Reviewer #2:

Comment 2-1)

The authors wrote that all sequence data has been uploaded to DDBJ and is waiting for an accession number to be issued. In addition, there are no statement regarding code availability. Please make sure to make all data and computational code underlying the findings fully available.

Reply 2-1)

We have made the sequencing data open on October 15, 2024 (now “ready for release” status), which can be downloaded from the DNA Data Bank of Japan (DDBJ) with accession numbers PRJDB18166 and PRJDB18259.

Also, we have made the numerical codes, which are now available from https://osf.io/6gtuy/. This information is described in the section of "data and code availability". Related to this revision, the starting sentences in section of Material and Methods are also altered.

We would like to thank again the reviewers for the comments that have helped us improve this manuscript.
