# Supplementary material for: Integrative analysis of ATAC-seq and RNA-seq for cells infected by human T-cell leukemia virus type 1
Source: PLoS Comput Biol. 2025 Jan 2;21(1):e1012690. doi: 10.1371/journal.pcbi.1012690 (PMC11753684; doi:10.1371/journal.pcbi.1012690)
Supplement: Attachment — Submitted filename: Reply_241125.pdf [file pcbi.1012690.s004.pdf]

Dear reviewers,

Thank you very much for your constructive comments and accurate suggestions on our manuscript. Following the comments, we hereby resubmit our revised manuscript.
Thank you once again for your consideration of our paper.

Sincerely,

Azusa Tanaka, Yasuhiro Ishitsuka, Hiroki Ohta, Norihiro Takenouchi, Masanori Nakagawa, Ki-Ryang Koh, Chiho Onish, Hiromitsu Tanaka, Akihiro Fujimoto, Jun-ichirou Yasunaga, Masao Matsuoka

****

Reviewer #1:

The authors have now properly responded to my comments and the mansucript is worth to be published. I'd like to confirm one point whether the ATL patients analyzed in this study are aggressive ATL patients. If so, the authors should describe acute type ATL patients instead of ATL patients in MATERIALS AND METHODS part. In acute type ATL patients, most of the cells sorted by Flow cytometry as CD4+/CADM1+ are tumor cells.

Reply)

We appreciate your comments. The samples used in this paper include not only acute but also chronic samples, and the details are described in Table 2. In response to your comments, we have added clinical information regarding the samples used for real-time PCR in Table 6, in the "Materials and Methods" section.

We would like to thank again the reviewers for the comments that have helped us improve this manuscript.
