# Supplementary material for: Integrative analysis of ATAC-seq and RNA-seq for cells infected by human T-cell leukemia virus type 1
Source: PLoS Comput Biol. 2025 Jan 2;21(1):e1012690. doi: 10.1371/journal.pcbi.1012690 (PMC11753684; doi:10.1371/journal.pcbi.1012690)
Supplement: Attachment — Submitted filename: 137664_1_rebuttal_2895455_s6qzwg.docx [file pcbi.1012690.s001.docx]

**Manuscript number:** RC-2023-02059

**Corresponding author(s):** Azusa Tanaka, Yasuhiro Ishitsuka and Hiroki Ohta

## 1. General Statements [optional]

Thank you for handling with our manuscript. Below, we have carefully replied to the comments by the two referees and have made the corresponding revisions.

Major revisions are done as responses to comment 1-1 (related to Fig. 13), 1-4 (section 2-F), 1-5 (section 2-F, 2-H, and Discussion), 1-7 (Discussion), 2-1 (Fig. 9), and 2-2 (Fig. 8). All the revisions, including others, are highlighted with red in the manuscript.

## 2. Point-by-point description of the revisions

**[Referee 1]**

[Comment 1-1]

Data from multiple clinical specimens were obtained, but subsequent analysis was done by randomly taking reads from each group and analyzing them together; ATL and HAM/TSP are heterogeneous diseases because of the individuality of the patients, and differences between patients should be considered. This study should be clustered as a whole with individual data, not an averaged data analysis.

[Our reply 1-1]

It is true that we did "randomly taking reads from each group and analyzing them together" as a part of our analysis, which is used in Fig. 1, Fig. 2, Fig. 4, Fig. 5, and Fig. 12. The procedure of "randomly taking reads from each group and analyzing them together" has been done to determine which locations in chromatin are effective to characterize "each sample" of ATAC-seq data. This is one of the methods used to analyze ATAC-seq data in previous papers [Nat. Genet. 2016; 48(10): 1193-203, Cancer Cell. 2017; 32(1): 27-41.e4.].

In Fig. 1, Fig. 2, Fig. 4, and Fig. 12, we focused on only the averaged property. In Fig. 5, we focused on heterogeneous properties of samples. Thus, our analysis shown in Fig. 5 provides a quantitative way to characterize the heterogeneous properties of ATL and HAM samples.

Furthermore, as for Table 1, we did not perform "randomly taking reads from each group and analyzing them together". Instead of doing it, we analyzed the ATAC-seq data from the perspective of how the cells in each patient sample are classified. This is not an averaged quantity at all. Thus, we have studied the quantities which characterize heterogeneous properties of samples as well.

[Revision 1-1]

We have added the following sentence to the end of section 4-B.

"This procedure is schematically described as a part of Fig. 13."

[Comment 1-2]

In all analyses, only mathematical data are handled, and no clinical interpretation or biological considerations are made. Algorithmic analysis can be effective in analyzing big data, but data analysis alone does not reach an understanding of the disease. Because it does not include any biological considerations, there are few new discoveries about disease from valuable clinical specimens.

[Our reply 1-2]

In order to take a solid step toward understanding the disease, we have mainly focused on reliable and verifiable results that, in principle, can be calculated by anyone who shares ATAC-seq and RNA-seq data in the paper. Please also refer to 3) in [Our reply 1-8].

Further, we had already given clinical interpretations and biological considerations in the discussion. One of them is about CTCL samples, which is discussed again in [Our reply 1-7]. Additionally, the sixth paragraph in discussion is about biological consideration.

It is one of our goals to facilitate the next research for more discoveries based on the very basic statistical analysis performed in this paper. In our opinion, the results of this paper are worth publishing to advance our knowledge towards a complete cure for the disease.

[Comment 1-3]

The present data show that there are differences based on chromatin structure, but they do not clearly show how they differ, and there is a lack of understanding of whether chromatin conformational changes are significant for the disease.

[Our reply 1-3]

ATAC-seq is a method to analyze chromatin structure on a genome-wide scale, and therefore the amount of information is enormous. As the referee pointed out, we are not looking at the differences in each of 3 billion base pairs. Instead, we took 5 different viewpoints to study how chromatin structures differ among CD4+T, HAM, and ATL in Fig. 1, Fig. 2, Fig. 3, Fig. 4, and Fig. 5.

We also used genome-wide open chromatin information to classify ATL samples based on our previously developed method. This classification result shown in Table 1 provides a viewpoint of how the chromatin structure of each sample differs. Those results are obviously new knowledge about the chromatin structures of ATL and HAM in comparison with that of CD4+T.

As is well known, ATAC-seq does not provide complete information on the three-dimensional structure of chromatin. In other words, since we use only ATAC-seq to obtain the information about chromatin structures, it is not feasible for us to study the full range of 3D structural changes of chromatin occurring in cells. With the help of other technologies such as Hi-C or single cell data, one could study in more detail of chromatin structures, which is not the target of this study.

It is not easy to experimentally verify how chromatin conformational changes are involved in disease. dCas9-KRAB, dCas9-VP64, and other technologies can be used to induce local chromatin conformational changes, but it is not feasible for current technology to experimentally induce genome-wide conformational changes. The latter is the case that we focused on in this paper. Thus, this paper does not attempt to quantitatively evaluate the significance of chromatin structural changes in specific regions to the onset of ATL.

[Comment 1-4]

It is not clear why you chose TLL1. What other genes were present need to be brought out well with heatmap, etc., to show what the overall cellular change is due to the chromatin structural change.

[Our reply 1-4]

We had already explained why we chose TLL1 by our method in section 2-G. To summarize the procedure, along changing M of the top M peaks obtained from ATAC-seq, we pick up the genes which have overlaps with, at least, a part of M peaks from ATL samples but do not have overlaps with all the M peaks from all the samples of 13 types of hematopoietic cells. Then, we found TLL1 one of the selected genes; the other selected genes except for TNRC6A were already reported in previous works, and then TLL1 had never been reported in this context. Thus, we decided to analyze TLL1 in the detail.

Following the referee comment, additionally, we have elaborated on how chromatin changes in the region of TRA mentioned in Fig. 9. Then, we have found that ATL samples show large sample-to-sample fluctuations in TRA region, which is consistent to Fig. 5.

Of course, the functions of other genes such as more than 20000 genes besides TLL1 and TRA are of interest, but those are not the target of this study. Again, as explained in [Our reply 1-3], only ATAC-seq does not allow us to have the complete information about conformational changes of chromatin. It is above the target of this study.

[revision 1-4]

We have added three paragraphs to discuss chromatin accessibility in TRA region to section 2-F, which are the second and the third paragraphs in the manuscript.

[Comment 1-5]

For TLL1 isoform, it is possible to identify which one is actually expressed from the RNA-seq data. The TLL1 actually expressed in ATL needs to be analyzed to determine what effect it has on TGF-b. Experimental verification of how this affects the nature and pathogenesis of ATL is also needed. Chromatin conformational changes in TLL1 have also been observed in HAM. It is necessary to consider whether the effects of TGF are also likely to be affected in HAM.

[Our reply 1-5]

Thank you very much for pointing out this important topic. As the referee pointed out, to get a deeper understanding of ATL, it is necessary to identify which isoform of TLL1 is dominantly expressed from RNA-seq data.

Following to the comment, we re-analyzed RNA-seq data by RSEM using "rsem-calculate-expression" command and found that the expression level of TLL1 isoform1 is about 10.3 times higher than that of TLL1 isoform2.

We also agree with the referee comment about the effect of each isoform of TLL1 on ATL pathogenesis. Although we have not shown the data in this paper, we have examined the effect of TLL1 on viral transmission by using p19 (viral protein) enzyme-linked immunosorbent assay (ELISA). Furthermore, we have performed Luciferase Immunoprecipitation System (LIPS) Assay to investigate the presence of TLL1 antibody in plasma derived from ATL patients. Then, there were no results indicating that TLL1 affects the infectivity of HTLV-1. Also, no TLL1 antibodies were identified in plasma derived from ATL patients.

This may be due to the fact that only the short isoform, TLL1 isoform2, was used in these experiments at the time they were conducted. Indeed, the above observations for the experiments with ELISA and LISP are consistent with our new analysis by RSEM that the expression level of TLL1 isoform 2 is zero or very low in each sample, which is also already mentioned above.

We plan to reexamine these experiments using TLL1 isoform1, which is by far the most abundantly expressed isoform. We would like to accumulate more data on this topic, which we hope to present as our next paper.

As for the ATAC-seq samples of HAM, we have added new classification results for individual samples of HAM to section 2-F, along with the classification results for individual samples of ATL that we have already done (Table 1).

As for RNA-seq, we were not allowed to analyze HAM samples because of the following situation; first, the number of cells that could be collected by flow cytometry was very small due to the low percentage of HTLV1-infected cells in the HAM cases. Since we had to use all the collected HTLV1-infected cells to obtain sufficient amount of ATAC-seq data, we could not perform RNA-seq in HAM cases. We will consider the analysis of RNA-seq data from HAM cases as the future study when the samples are ready.

[Revision 1-5]

We have added the following paragraph to section 2-H.

"Taking into account the role of TGF-beta in cancer progression, the above observations suggest that TLL1 isoform 1 is more expressed in ATL samples, compared to TLL isoform 2. Indeed, we analyzed again the RNA-seq of ATL samples and confirmed that the expression of TLL1 isoform 1 is about 10.3 times larger than that of TLL isoform 2 on the average over 4 samples (see section IV C of Material and Methods section for the detail). Moreover, TLL1 isoform 2 is not detected in 2 samples out of 4 samples."

We have added the following sentence to the third paragraph of discussion.

"Indeed, the RNA-seq data of ATL samples imply that activation effect by TLL1 isoform1 (NM_012464) on TGF-beta is dominant over repression effect by TLL1 isoform2 (NM_001204760)."

We have added the following sentence to section 2-F.

"Note that by using the same method as that for ATL samples, ATAC-seq data of each HAM samples are classified into CD4+T as the first, CD8+T as the second, NK as the third. Thus, the classification of the HAM samples by chromatin structures does not depend on the samples."

We have added the following sentence to the fourth paragraph of discussion.

"Note that, due to the lack of proper RNA-seq data of HAM samples, we were unable to study gene expression of HAM samples, which remain to be analyzed in future studies."

[Comment 1-6]

Although footprint and other data are only included in the discussion, these data are subject to evaluation and must be included in addition to the results section.

[Our reply 1-6]

We appreciate the supportive comments from the referee. The reason why we have included the results of the footprint analysis is because we would like to suggest that the transcription factors that function in ATL cases may be altered from CD4+ T cells from healthy individuals. We believe that more analysis such as ChIP-seq and knockdown experiments need to be performed to understand what the footprint analysis exactly indicate. Further, the current version of the footprint analysis is independent of the main theme in this paper. Thus, we keep this in discussion. We believe this is one of the important themes for the future.

[Comment 1-7]

You cited CTCL data, but the discussion is confusing.

[Our reply 1-7]

This is a part of clinical interpretation or biological consideration including our speculation, which can be an answer to the referee's comment in [comment 1-2].

We classified the chromatin structure of CTCL cases both before and after the treatment with HDAC inhibitors using a method developed in our previous paper. The results suggest that there is a relationship between the drug treatment responsiveness and pre-treatment chromatin structure. This results on CTCL is interesting because it might imply that chromatin structures provide useful information for patient segmentation and selection of cancer treatment.

We used the data from CTCL because of the following two reasons. First, although we obtained ATAC-seq data of pre-treatment samples, we could not obtain ATAC-seq data of the post-treatment samples. Second, CTCL is a T cell leukemia whose pathology is very similar to that of ATL. By this similarity, we expected that the relationship observed in CTCL might appear in ATL cases. However, this is still a level of speculation. We have an option to remove this part. Note that this part in itself does not affect the main results of this paper.

[Revision 1-7]

We have replaced the sentence "the change in immunophenotype reflects a molecular response in treatment" written in discussion by the sentence "the types of ATAC-seq characterized by our method could be strongly correlated with whether the patient can recover after the drug treatment".

[Comment 1-8]

The current study by Tanaka et al. focused on ATL and HAM/TSP, two HTLV-1-associated diseases with poor prognosis, and examined changes in chromatin structure by ATAC-seq. The analysis was performed on many clinical specimens and included information of both basic and clinical importance. However, the authors rely on a mathematical approach and analysis of existing algorithms, which do not provide direct information about the pathophysiology and mechanisms of disease. They also do not take into account the differences that exist between patients and analyze the data by integrating them, thus showing only common and slight differences. The analysis is not based on the accepted methods of general ATAC-seq analysis, making it extremely difficult to read and significantly reducing the value of the clinical specimens.

Although the value of the ATAC-seq data is high, the lack of integrated data analysis and lack of clinical interpretation seriously impair the quality of the study. Without overall improvement, the study has not reached the level of publication as a paper.

[Our reply 1-8]

First of all, as explained by the whole of our replies and revises, we have corrected the parts of our paper which potentially misled the referee's evaluation described by all the above comments. Thus, we believe that this last comment will be updated after taking into account all of our replies and revises. Below, we additionally remark on the following three parts of the last comment.

1) The comment says that "They also do not take into account the differences that exist between patients and analyze the data by integrating them". We do not really agree with this description. Indeed, we have analyzed how sample-to-sample fluctuations in ATAC-seq data behave in section 2-C. This quantity certainly characterizes the differences that exist between patients. Further, we have analyzed ATAC-seq data to classify the corresponding chromatin accessibility for each patient as shown in Table 1, which is not an averaged quantity at all. As pointed out by the referee, we did not analyze differences that exist between patients in RNA-seq because we do not have enough number of samples. That is a future study when such samples get ready.

2) The comment says that "The analysis is not based on the accepted methods of general ATAC-seq analysis". Unfortunately, we are not convinced of what "accepted" in the comment means. Our analysis relies on the very basic statistical quantities such as average, variance, histogram, etc as compactly described in Material and Methods including the standard pre-analysis such as MACS2 algorithm developed by [Zhang, Liu, Meyer et al., Genome Biol. 2008]. Those are definitely accepted methods in statistics and bioinformatics related to genetics. Further, our algorithm developed in the previous work has been literally accepted as an article [Tanaka, Ishitsuka, Ohta, et al., PLOS Comput. Biol. 2020].

3) The comment says that "the lack of integrated data analysis and lack of clinical interpretation seriously impair the quality of the study". Contrary to this description, first, we did perform an integrated analysis of ATAC-seq and RNA-seq in section 2-E, which was shown as Fig. 7. Second, we did provide a clinical interpretation in the discussion as already mentioned in our reply [reply 1-7]. Apart from these two facts, we have carefully checked the very basic statistical properties of ATAC-seq and RNA-seq by avoiding black-box types of advanced algorithm as much as we could. Then, we found nontrivial and elusive statistical properties, which could be overlooked. In this sense, our analyses have partly succeeded in reaching the elusive information given by patients.

We believe that this kind of basic analysis as we did in this paper should be done along with the analysis using the other accepted methods including more advanced algorithm, which often have packaged versions for application. Such advanced analyses can be done as other works by professional researchers of each method. The ATAC-seq data we have analyzed is going to be open-access along this paper, meaning that we do not "impair" anything. Note that a further experimental study about TLL1 isoform 1 that our work did not perform is undergoing as another work by some of the authors of this paper.

**[Referee 2]**

[Comment 2-1]

The technical procedure to obtain Fig. 9 is not clear. I did not understand where/which the authors used their previous method. In addition, if M means the reason for top M peaks, it seems that the number of genes monotonically increases, which is not consistent with the figure. Moreover, the numbers of genes in the manuscript and figure do not coincide. I request them to add an explanation of this section carefully.

[Our reply 2-1]

As the referee pointed out, the caption in Fig. 9 has not been well described although the explanation has been already written in the second paragraph in section G.

As pointed out by the referee, M is the M of the top M peaks obtained by MACS algorithm from ATAC-seq data. Then, we selected the genes that 1) have overlaps with, at least, a part of the M peaks from ATL samples and also 2) do not have overlaps with all the M peaks from any sample of 13 types of healthy hematopoietic cells. Thus, only the genes satisfying the two conditions were selected. That is the reason why the number of selected genes does not necessarily increase monotonically.

Further, in Fig. 9b, we have shown only the part of the selected genes, which stay selected after more than 8000 increment for the value of M.

[Revision 2-1]

We have replaced the caption by new one totally.

[Comment 2-2]

Why do you need to divide by read counts in TRA in Fig 8? It seems inconsistent with the explanation of the manuscript.

[Our reply 2-2]

In fact, read counts trivially depend on the amount of input or setting of sequencers. Thus, in order to reasonably compare different samples, we have to somehow normalize the read counts in TREC region. That was the reason why we divided it by the read counts in TRA gene coding region, which includes TREC region. However, thanks to the referee's comment, we have realized that the read counts in TRA region could also depend on cell types even if the amount of input are the same.

Then, we have examined the property of the top 64000 peaks obtained by MACS2 algorithm in the regions of TREC and TRA. It is because those top peaks do not strongly depend on the amount of input and are relevant to cell types as our previous study showed.

As expected, among CD4+T and ATL, the total width of peaks in TREC region are 0. Additionally, that of CD8+T, HAM out of the other types are the two smallest on the average. This is consistent to the previous observation that HAM and ATL show the immunophenotype of the memory T cell, where gene reconstruction is made at TREC region. The ratio between the total width of peaks in TREC and that in TRA also gives additional evidence to support the same conclusion.

Note that some samples of Ery also show 0 peak in TREC region. This is not contradiction with the above observation because 0 peak in the case of Ery just implies that chromatin structures of TREC region in Ery tend to be closed. This is not related to the main topic.

[Revision 2-2]

We have made new figures as Fig. 8 and also modified the caption of Fig. 8 totally. Note that [revision 1-4] is also related to this part.

[Comment 2-3]

I think the use of the word "reads counts" is not common instead of "read counts".

[Our reply 2-3]

Thank you for informing us of it. We have revised all the corresponding parts.

[Comment 2-4]

Correct mathematical environments such as M>8000 in P. 11.

[Our reply 2-4]

Thank you for pointing out it. We have modified the font of the corresponding part.

[Comment 2-5]

Include correct funding information.

[Our reply 2-5]

Thank you for pointing out it. We have added the correct information about funding to Acknowledgement.

[Comment 2-6]

The manuscript presented a way for when, where, or how we should use/analyze bulk ATAC-seq and RNA-seq data. This paper succeeded in narrowing the candidates of significant genes down because they focused on mutation by disease. In contrast, their method may be difficult to extend to more diverse data such as single-cell omics.

In addition, it is noticed that this paper is irrelevant to the data integration of multi-omics because each analysis independently uses ATAC-seq or RNA-seq data.

My expertise is in data science and bioinformatics. Hence I could not evaluate the impact of this paper on biology.

[Our reply 2-6]

Thank you for understanding our achievement. As for the extension from our algorithm to single-cell omics, obviously it is not straightforward to make it. We need additional ideas to adjust with single-cell omics, which will be a target of future studies.

As for the integration of ATAC-seq and RNA-seq for which the referee regards our study as irrelevant, indeed we have shown one way to integrate the two omics data in section 2-E; Concretely, the orange lines in Fig. 7 correspond to the parts of RNA-seq data selected by ATAC-seq data with parameter M equal to infinity. Blue lines in Fig. 7 correspond to the parts of RNA-seq data selected by ATAC-seq data with parameter M equal to 64000. This is exactly one way by which we can see clear correlations between ATAC-seq and RNA-seq. Before section 2-E, we have analyzed each of two data independently in the sections from 2-A to 2-D, which is exactly pointed out by the referee; Concretely, we focus on only ATAC-seq in section 2-A, 2-B, and 2-C whereas we focus on only RNA-seq in section 2-D.
